# Diminished psychedelic returns on distress: Marital status and household size

**DOI:** 10.1371/journal.pone.0293675

**Published:** 2024-03-07

**Authors:** Sean M. Viña

**Affiliations:** Department of Sociology, The University of the Incarnate Word, San Antonio, Texas, United States of America; University of Botswana, BOTSWANA

## Abstract

Although the use of psychedelics to impact health has seen growth, little research has tested the effects of culture conditions on the relationship. More specifically, how does marital status and family size affect the relationship between psychedelics and health? This study tests the relationship between Lifetime Classic Psychedelic Use (LCPU), marital status, and household size (number of people living in a household) on levels of psychological distress in the past 30 days. This project uses pooled data from the National Survey of Drug Use and Health (NSDUH) (2010 to 2018) (*N* = 674,521). The Final sample size is determined by the dependent variable, psychological distress in the past month (n = 158,633). The analysis includes a series of nested logistic regression models conducted in Stata 17. Results indicate that LCPU is independently associated with better health, but the association between LCPU and health varies across levels of household size. Larger households are associated with higher levels of distress, which are then exacerbated among psychedelics users. Furthermore, three-way interactions reveal that the negative association between household size and distress gets larger among psychedelic users who are married, divorced, and widowed. Overall, results suggest that household size negatively impacts the association between LCPU and health, with those who are married, divorced, and widowed experiencing the worst outcomes.

## Introduction

Research has found a positive relationship between psychedelics and health. Clinical trials have found robust positive effects of psychedelic-assisted therapy in treating health problems, including depression, anxiety, suicidality, PTSD, drug dependency, and other behavioral addictions [1–14]. Some of the most promising studies have found that one dose of psychedelic-assisted treatment can effectively eliminate major psychological problems that years of other therapy and pharmaceuticals were unable to treat [15–17]. Additionally, Populations studies have confirmed mainly clinical research showing that various psychedelics and 3,4-methylenedioxymethamphetamine (MDMA), commonly known as “ecstasy,” are associated with better physical health and mental health as well as with decreasing negative social behaviors, including committing larceny and domestic violence [3, 18–30].

However, growing evidence suggests that the protective effects of psychedelics differ among sub-populations. National data has shown that psilocybin and MDMA use was linked to improved health outcomes for white users. However, there were minimal or no associations observed among users from different racial or ethnic minority groups [31, 32]. Another study found that, while those who are employed and use psychedelics have significantly lower levels of psychological distress, non-employed users have higher levels of distress [33]. Another study found that while Black psychedelic users saw no decrease in stress, white psychedelic users saw a significant increase in stress, which was further amplified by education [34]. Additionally, their study found that criminal history significantly attenuated the positive association of psychedelics for white users, but not black users. Another study documented how religious beliefs can interact with psychedelics, affecting mental health. The study found that psychedelics can interact with strong religious beliefs, creating better health outcomes, but only when religious attendance was also high [35]. Those who had strong beliefs but low attendance had higher levels of distress. The findings suggest that culture (levels of religiosity) and social interactions (amount of religious attendance) alter set and setting, which affects psychedelics’ impact on health.

A modified version theory cultural set-and-setting, which takes a social-epidemiological approach, has been proposed as an explanation to identify smaller health gains of psychedelic use among minority populations [34]. The first iteration of the theory of cultural set-and-setting proposed that because the effects of psychedelics are “fundamentally reliant on context–both in the psychological and environmental sense” [36], cultural conditions can impact the overall effectiveness of psychedelic on health. Specifically, setting refers to the physical and social environment where psychedelics are consumed. In contrast, set “refers to the internal conditions of the person using the psychedelics, including factors such as mood, attitudes, preparation, personal history, personality, expectations, motivations for using, and beliefs about themselves and the use of drugs” [37]. According to Hartogsohn [38], the individual level where the psychedelic is consumed sits “atop of a more fundamental collective level, which frames and gives shape” to set-and-setting available to the.

Although focused on issues of race inequality, the modified cultural set-and-setting theoretical framework expands to include structural inequalities that may further negatively impact the sequelae of psychedelics on health beyond the cultural interpretation during use. Researchers theorize that systemic inequality will affect the entire relationship that people have with psychedelics, both upstream before use and downstream after use [34]. Specifically, while initial researchers proposed that cultural conditions could affect the perceptions of psychedelics during use [38], the modified theory suggests that structural inequalities could also affect access to other resources or exposure to trauma/stress post psychedelic use which more quickly diminishes the efficacy of the drugs [34]. To be sure, different social groups (i.e., gender and socioeconomic status (SES)) have access to different types of resources that will affect set-and- setting. Moreover, cultures and subcultures provide “distinct types of environments for hallucinogenic experimentation” [38]. With their focus on race, the modified theory suggests that the diminished psychedelic returns among race and ethnic minorities is likely explained by pervasive and everyday mistreatment which negatively impacts other health conditions including worse allostatic load, multisystem index of biological dysregulation [39, 40], poorer sleep duration and efficiency [41], greater systemic inflammation [42], a greater prevalence of distress and mental illness, worse negative coping behaviors such as smoking [43], and worse late life cognitive function [44].

Drawing on the modified theoretical framework, this paper proposes that, due to socio-cultural differences and potential inequalities, we should anticipate diminished or varied psychedelic outcomes for certain subpopulations, particularly in relation to marital status and household size. To be more precise, individuals who engage in psychedelic experiences (referred to as "psychonaughts") appear to achieve the most positive health outcomes when they are in a supportive and trusting relationship with their counselors in a clinical setting [45]. Alternatively, positive outcomes can also be observed within an empathetic and supportive community in a naturalistic environment [46]. Therefore, due to factors such as the structure of the family, the strength of relationships, and variations in familial responsibilities, individuals from different groups may have diverse outcomes after using psychedelics. These factors can influence how someone interacts with psychedelics and interprets their experiences. It is important to acknowledge that these differences exist.

This study utilized data from the National Survey of Drug Use (N = 2008 to 2019), with a total of 674,521 participants. The findings of the study reveal significant interactions between marital status, household size, and lifetime classic psychedelic use (LCPU) in relation to levels of distress. The results indicate that LCPU amplifies the negative impact of household size on distress. Specifically, for each additional person in a household, there is an increase in distress levels, and this increase becomes more pronounced at a higher rate. Conversely, for individuals who have not used psychedelics, larger households are associated with more distress, but the association diminishes at a decreasing rate. Although there is no direct interaction between LCPU and marital status on health, the study’s three-way interactions demonstrate that household size influences the relationship between LCPU and marital status. Larger households generally contribute to more distress, especially among married and divorced psychedelic users. However, widowed psychedelic users experience some benefits from increased household size, but only within a smaller household. The implications of these findings are discussed in more detail below.

## Literature

### Marital status and family sizes effect on psychedelic returns

To understand how marital status and household size may interact with psychedelics, it is important to highlight a theoretical point on the modified cultural set-and-setting theory. The modified theory of cultural set-and-setting recognizes two key definitions. "Setting" refers to the physical and social environment where psychedelics are consumed. In contrast, "set" refers to the internal conditions of the person using the psychedelics, including factors such as mood, attitudes, preparation, personal history, personality, expectations, motivations for using, and beliefs about themselves and the use of drugs [47]. Overall, the theory hypothesizes that set and setting sit on a spectrum, ranging from the most optimal to the most compromised. Individuals with the most optimal set and setting conditions, including psychological, biological, and social factors, are more likely to experience sustained health outcomes [33, 34]. Conversely, individuals who have a compromised set and setting have fewer of the necessary conditions to achieve optimal health outcomes related to psychedelic use. It is crucial to note that sociocultural and structural differences will influence various aspects of set-and-setting, placing people at different points along the spectrum.

In the case of marital status and family size, issues of social integration may be particularly salient for the positive effects of psychedelics on health. Social integration, which refers to the involvement in social relationships and social contexts [48], has been hypothesized to have multiple dimensions. These dimensions include the prominence or the degree to which a person is committed to or embedded in a relationship or group based on their roles, the salience or the intensity and extent of social ties, the emotional charge of relationships, the strength of ties in providing resources, and the availability or access to alternative networks [49]. Importantly, increasing social integration can have both positive and negative health outcomes. For instance, some evidence suggests that having larger and more supportive networks can lead to better health outcomes and longer life expectancy due to the availability of resources [48, 50], negative social integration such as the spread of emotion or ideas as well as increased social expectations can lead to higher distress, suicide, and negative coping strategies in relation to HIV health promotion [49, 51–55].

Most importantly, positive social integration has long been recognized as a critical component of the psychedelic experience by both clinical and cultural researchers. The success of psychedelics relies on the positive meaning-making that occurs in these socially controlled situations [56]. Psychedelics appear to stimulate creative thinking, enabling individuals stuck in negative thought patterns to explore new and positive avenues of understanding that were previously inaccessible due to their mental illness. Before receiving drugs in clinical settings, participants undergo comprehensive psychological counseling. This counseling aims to identify sources of stress, foster a positive rapport with counselors, and create a trusting and secure environment [7]. The importance of social integration is not surprising, as anthropological research on psychedelics has consistently shown that historically, these drugs were consumed in communal religious settings within various indigenous societies. Examples include Bwiti in Africa, the Amazon tribes in South America, the Aztecs in North America, and the Mayan and Inca in Central America [11, 57–59]. Contemporary practices have tried to replicate those communal settings by consuming psychedelics in natural settings within a collective group [46]. The saltatory relationship between psychedelics and positive social integration is not surprising, as classic Durkheimian theory of social integration has found robust evidence for a consistent relationship between social support and health [48, 60, 61].

Why marital status is likely to affect the relationship between psychedelics and health is because of different aspects of social integration. The evidence shows that psychedelics are more effective in comfortable settings with a supportive guide, group, or counselor [45]. Several studies have found that, while psychedelics can create an intense feeling of openness and empathy, the most fulfilling psychedelic experiences were those that drew upon personal experiences or relationships [56]. Thus, it is likely that the reason positive social integration interacts with psychedelic experiences is because not only do psychedelics boost feelings of empathy and connectedness, but those who are more positively socially integrated may also have more tangible enacted resources [57]. This allows them to experience firsthand the perception that they are loved and cared for, critical for improved health [58].

However, ample research has found that different marital statuses have different levels and types of social integration. For instance, compared to singles, cohabitors, divorces, and those who are widowed, married people have a more supportive environment and greater social and psychological resources [59–68]. More specifically, married people have the largest and most supportive networks, which leads to increased positive engagement. This is why those who are married have a lower risk of cardiovascular disease, mortality, and longer life expectancy [69–71]. Network analysis also suggests that married people tend to have larger and more supportive social circles, which extend further through their spouse networks [72–74]. More specifically, those who enter a relationship, particularly those who enter marriage, instantly gain access to their spouse’s networks, which can improve their social support and sense of belonging.

But, while it is likely that marriage is associated with many more tangible resources, the modified theory stresses the interplay of cultural conceptions. Thus, we must consider the stigma attached to drug use [75]. Moreover, social integration can have negative effects when it comes to role expectations and the availability of alternative identities. For instance, individuals who are not in a romantic relationship or are single are more likely to experience social isolation, which in turn reduces their chances of having a supportive relationship that can positively impact their psychedelic experiences. On the other hand, those who are married and may have a support system also face different expectations regarding their roles within their families. Additionally, there are cultural differences in the perception of drug use, especially among parents. Despite the growing popularity of psychedelics, they still largely remain taboo. A mixed-methods meta-analysis revealed that while drug use is stigmatized across various substances including marijuana, heroin, cocaine, amphetamines, and alcohol, those who were married and had family responsibilities faced particularly strong stigmatization [76]. And even though there have been dramatic shifts in psychedelic public acceptance, stigma is likely to remain high, as demonstrated by marijuana. Although marijuana use has increased dramatically in the last few decades, the vast majority of marijuana users report experiencing discrimination attached to their use. This is possibly because at least 40% of Americans believe that marijuana use is morally wrong [77, 78]. Most importantly, evidence suggests that there are higher rates of stigma attached to those who are married or living with children [79]. Thus, it is likely that negative conceptions attached to psychedelic use may lead to less support and fewer opportunities to positively engage with psychedelics among those who are married.

Additionally, while marital status is likely to affect psychedelics and health, we have to remember that not all family structures are the same. Ample evidence has found that larger families are associated with higher levels of stress [80–82]. More recently, evidence has shown that larger households contribute to an increased risk of distress during COVID-19 pandemic lockdowns [83]. Most importantly, the level of distress is dependent on the relationship with those structures, or the quality of social integration. Larger families seem to produce their most negative consequences for those who are heads of households, which is explained by an aggregation of responsibilities and expectations to devote time and resources to the family [81]. With larger families, there is an increase in responsibility, including childcare, schooling, cooking, and household management. While some of these tasks can be shared with a partner, research shows that these responsibilities can have a negative impact on larger families. One of the primary reasons for this is the financial strain that comes with having a larger family. As the family size increases, so does the financial burden. This burden is particularly challenging for individuals who are divorced or widowed, as they may have lost the support of social connections that could have helped alleviate some of the strain [84–87]. The increase in stress following a recent divorce or the death of a partner, which results in increased familial responsibility, partially explains their heightened risk of suicide and substance abuse [85]. Thus, while married people may have more negative social integration because of familial expectations, they may also have more positive social resources. On the other hand, individuals who are widowed or divorced may still have familial responsibilities, but with even fewer resources to meet those demands.

Overall, higher levels of distress and fewer resources may lead to a faster decrease in the efficacy of psychedelics for heads of households, especially those who are widowed or divorced. Stigma may also have a compounding effect on household size, as heads of households may feel more guilt about using psychedelics as their familial responsibilities increase. On the other hand, single individuals are less likely to be heads of households, so as family sizes grow, they may experience less stress since the responsibility can be shared among the group. It is crucial to note that psychedelics are a reflection of social circumstances, and the link between marital status, household size, and distress may be intensified by low community psychosocial unity (LCPU).

## Empirical predication

This study examines the relationship between psychedelics, marital status, household size, and distress. It draws upon a modified theory of cultural set-and-setting, as well as a long history of theory and research in the stress process tradition [88]. The research predicts several relationships between the aforementioned resources and psychological distress. The respective predictions highlight potential relationships that are conceptually distinct but not mutually exclusive. First, it predicts that marriage and psychedelic use will be associated with less distress. Second, the study predicts that larger households will be associated with a higher level of distress. Third, due to higher levels of distress and fewer social/psychological resources, the study predicts that the association of psychedelics on distress will be proportionally smallest among people who live in larger households. In other words, the positive association of LCPU on distress will be diminished among larger households. Fourth, because of the availability of ample social and psychological resources, the study posits that those who are married may gain the most benefit from psychedelic use. Specifically, the magnitude of this association between psychedelics and distress will be proportionally smaller among married individuals. Finally, the study predicts that the association between household size, psychedelics, and distress will vary by marital status, due to differences in familial responsibilities and available social support. Specifically, the negative association between household size and distress will worsen for individuals who are the heads of their households, likely those who are married, divorced, and widowed.

## Data and methods

This present study utilized pooled, cross-sectional data from the National Survey of Drug Use and Health (NSDUH) conducted from 2008 to 2019. The NSDUH is an annual survey conducted in all 50 states and the District of Columbia, with the aim of measuring the prevalence of substance use and mental health issues in the United States. The data was weighted to accurately represent the civilian noninstitutionalized population. Table 1 provides descriptive statistics for the dependent, independent, and control variables, all of which are derived from publicly available data. The NSDUH public-use data files can be accessed on their homepage: https://www.datafiles.samhsa.gov/dataset/nsduh-2002-2019-ds0001-nsduh-2002-2019-ds0001.

**Table 1:** Descriptive statistics for dependent variables, independent variables, and controls (2008–2019) (weighted).

	Mean	SD	n	% / min-max
Dependent Variable				
Psychological Distress in Last Month (K6)	9.58	6.08	161,573	0–24
LCPU			85,451	12.67
Independent Variables				
Marital Status				
Single			182,492	31.31
Married			|290,468	49.84
Widowed			33,017	5.67
Divorced/Separated			76,827	13.18
Household Size	3.12	1.45	674,521	1–6
Control Variables				
Women				
Age			347,420	51.51
Race				
White			435,982	64.64
Black			80,532	11.94
Native American			3,591	.53
Hawaiian			2,437	.36
Asian			|34,537	5.12
Multi-Racial			10,891	1.61
Hispanic			106,547	15.80
Educational Attainment				
Less than High School			66,274	27.83
High School			134,881	27.83
Some College			136,684	28.20
College Degree or Higher			146,891	30.30
Annual Household Income	4.97	2.02	674,521	1–7
Employed Full-Time			289,858	42.97
Religious Attendance	1.89	1.88	480,882	0–5
Religious Salience	4.92	2.60	472,653	0–9
Age of First Alcohol Use	1.64	.77	674,521	1–5
Lifetime Drug Use				
Tobacco			358,312	53.12
Cocaine			99,271	14.72
Stimulants			64,904	9.62
Sedatives			51,594	7.65
Tranquilizer			102,918	15.26
Inhalants			58,931	8.75
Pain Relievers			230,073	34.11
Heroine			12,119	1.80
Marijuana			294,133	43.61
PCP			16,102	2.39
MDMA/ecstasy			44,156	6.55
Self-Reported Risky Behavior	1.64	.77	670,710	1–4

Source: 2008–2019 National Survey of Drug Use and Health, *n = 674*,*521*

The NSDUH was originally conducted with approval from the Substance Abuse and Mental Health Services Administration’s internal review board. The original NSDUH obtained consent from participants based on their IRB guidelines. The publicly available data ensures complete confidentiality by removing all identifying information. Since this study is using anonymous, publicly available data, it did not need to seek ethics approval or obtain consent.

### Study replications

The present study replicates previous research that has analyzed the association between psychedelic use and health outcomes using the NSDUH [3, 18–20, 25, 27–29, 35]. While this paper replicates the study that analyzes the association between LCPU and distress, it aims to improve upon the previous research by addressing some of its limitations [3]. There are almost a dozen other papers that use an established statistical procedure for analyzing psychedelics and outcomes using the NSDUH, which this paper closely follows. This study uses the same dependent, independent, and control variables.

### Dependent variables

Respondents reported their level of distress in the past month using the Kessler Psychological Distress Scale (K6) [89, 90]. Participants indicate how often they have had six different feelings or experiences during the past 30 days using a 5-point Likert scale: 4 (All of the time), 3 (Most of the time), 2 (Some of the time), 1 (A little of the time), and 0 (None of the time). The feelings and experiences for this first item are the following: “nervous,” “hopeless,” “restless or fidgety,” “so depressed that nothing could cheer you up,” “that everything was an effort,” and “worthless.” Psychological distress in the past month created the variable by adding all measures into one scale that ranges from 0–24, with higher scores indicating more distress. The Kessler scale of psychological distress is a well-established, reliable, and valid measure of psychological distress in people (adults and adolescents across genders) with panic disorder, generalized anxiety disorder, bipolar disorder, and schizophrenia [91–93].

### Independent variables

The first independent variable is classic psychedelic use, a subclass of psychedelics that has little toxicity [14, 94, 95]. The three main classes of classic psychedelics—including tryptamines, lysergamides, and phenethylamines—are distinguished by unique chemical structures and neurochemical mechanisms [96]. Classic psychedelics include N-dimethyltryptamine (DMT), the DMT-containing admixture ayahuasca, psilocybin, lysergic acid diethylamide (LSD), mescaline, and the mescaline-containing cacti peyote. Respondents reported if they had ever used, even once, the following drugs: DMT, ayahuasca, LSD, mescaline, peyote, or psilocybin. Additionally, this study incldes of measure of MDMA because, although not a "classic psychedelic" because it induces a "flood of serotonin in the brain," while each of the classic psychedelics promotes neurogenesis [9], research finds that MDMA prodcues “psychedelic effect” which also are associated with a host of better mental health outcomes [4, 16, 27, 97–99] which is the process by which new neurons are formed in the brain Consistent with previous research [3, 18–20], the analysis uses a dummy variable indicating any lifetime classic psychedelic use (LCPU) (yes vs. no).

Marital status includes four categories: married, widowed, divorced/separated, and single (never married), with the last category being the reference. Household size is a continuous variable that measures the number of people living in a household, ranging from one to six or more.

### Control variables

Sociodemographic control variables include age (18, 19, 20, 21, 22–23, 24–25, 25–29, 30–34, 35–49, 50–64, and 65+), annual household income (less than $10,000, $10,000-$19,999, $20,000-$29,999, $30,000-$39,999, $40,000-$49,999, $50,000-$74,999, and $75,000 or more), and full-time employment status (versus not fully employed). The analysis includes multiple dummy variables: gender (women versus men), race/ethnicity (non-Hispanic African American, non-Hispanic Native American/Alaska Native, non-Hispanic Native Hawaiian/Pacific Islander, non-Hispanic Asian, non-Hispanic more than one race, Hispanic, and non-Hispanic white, serving as the reference category), and educational attainment (high school degree, some college, college degree or higher, and less than a high school degree, serving as the reference category). There are two continuous variables measuring religiosity. First, religious attendance is a continuous measure of how often a person attended religious services in the last year with the following options: (0 = ) 0 times, (1 = ) 1 to 2 times, (2 = ) 3–5 times, (3 = ) 6 to 24 times, (4 = ) 25 to 52 times, and (5 = ) more than 52 times. Respondents also indicated their level of agreement with the following three statements: (1) my religious beliefs are very important, (2) my religious beliefs influence my life, and (3) it’s important that I associate with religious people. These responses were summed to create a measure of religiosity ranging from 1 to 4 (Cronbach’s alpha = 0.84). The regression analysis also controls for the year of the survey, based on previous research using the NSDUH.

Previous research has found a strong correlation between drug use, risky behavior, and mental illness. Therefore, this study follows the guidance of similar research that uses the NSDUH to analyze the positive effects of psychedelics by including controls for other drug use that is negatively associated with poor health [3, 18–20, 25, 27–29]. Those binary control variables include lifetime use of cocaine; marijuana use; 3,4-methylenedioxymethamphetamine (MDMA/ecstasy); phencyclidine (PCP); inhalants; other stimulants; sedatives; pain relievers; and tobacco (smokeless tobacco, pipe tobacco, cigars, and daily cigarette use). Age of first alcohol use and self-reported engagement in risky behavior are continuous variables.

### Analytic strategy

To address the questions of this study, the first step was to calculate the mean of each variable for the four marital statuses: married, single, divorced, and widowed. Next, a post-estimation LINCOM (non-linear combination) command was used to compute the statistical difference between the means of two subpopulations [100], and calculated the statistical mean difference all dependent, independent, and control variables by the four marital statuses: married minus (-) single, married minus (-) divorced, married minus (-) widowed, and divorced minus (-) widowed (Table 2). I then used a series of ordinary least square regression models to test the relationship between marital status, household size, LCPU, and psychological distress over the past month (Table 3). I followed recommendation to run a sensitivity analysis of alternative models (i.e., probit, multinomial, and ordinary least square regression), but results proved substantially identical [101]. The first model predicts psychological distress with all controls. The second model introduces marital status and household size. Models 3 and 4 introduce interaction terms between LCPU and marital status and household size, respectively. Model 6 is the full model, which includes a three-way interaction between LCPU, marital status, and household size. Finally, it ran post-estimation Wald tests to estimate the inequality of two coefficients in regression analysis (i.e., singles vs. divorced or singles vs. widowed). I included the quadratic term for household size in the models to account for the possibility of a curvilinear relationship between household size and health. Finally, the analysis tests the marginal effects of the LCPU at each level point estimate of household size (1–6) to determine which levels remain significantly different in the interaction term [100].

**Table 2:** Means differences of key variables and control variables by marital status (weighted).

	Mean				Mean Difference ^a^							
	Married	Single	Widowed	Divorced	Married (–) Single		Married (–) Widowed		Married (–) Divorced		Widowed (–) Divorced	
Psychological Distress in Past Month (K6)	8.30	11.31	8.25	10.23	-3.01	^***^	.05		-1.91	^***^	-1.97	^***^
	(.038)	(.032)	(.116)	(.074)	(.050)		(.122)		(.083)		(.149)	
Lifetime Classic Psychedelic Use	.11	.14	.05	.18	-.02	^***^	.06	^***^	-.06	^***^	-.13	^***^
Household Size	3.21	3.29	1.92	2.42	-.07	^***^	1.29	^***^	.78	^***^	-.50	^***^
	.004	.006	.017	.009	(.007		.016		.010		.017	
Age	15.43	11.14	16.62	15.58	4.28	^***^	-1.19	^***^	-.15	^***^	1.04	^***^
	.004	.013	.006	.007	.014		.007		.008		.009	
Race												
White	.70	.54	.74	.65	.16	^***^	-.03	^***^	.05	^***^	.08	^***^
Black	.07	.17	.11	.14	-.10	^***^	-.04	^***^	-.07	^***^	-.02	^***^
Native American	.00	.01	.01	.01	-.00	^***^	-.00	^**^	-.00	^***^	-.00	
Hawaiian	.00	.00	.00	.00	-.00	^**^	.00	^*^	.00		-.00	^*^
Asian	.06	.05	.02	.02	.01	^***^	.03	^***^	.03	^***^	.00	
Multi-Racial	.01	.02	.01	.01	-.01	^***^	-.01	^**^	-.01	^***^	-.00	
Hispanic	.14	.19	.08	.14	-.05	^***^	.05	^***^	-.00		-.05	^***^
Educational Attainment												
Less than High School	.11	.15	.23	.15	-.03	^***^	-.08	^***^	-.00	^***^	.08	^***^
High School	.23	.29	.34	.30	-.04	^***^	-.09	^***^	-.05	^***^	.03	^***^
Some College	.26	.32	.22	.31	-.06	^***^	.03	^***^	-.04	^***^	-.08	^***^
College or Higher	.37	.23	.18	.22	.13	^***^	.18	^***^	.14	^***^	-.04	^***^
Annual Household Income	5.67	4.38	3.83	4.18	1.28	^***^	1.83	^***^	1.49	^***^	-.34	^***^
	.007	.009	.022	.012	.009	^***^	.023	^***^	.015	^***^	.025	^***^
Employed Full-Time	.51	.39	.14	.48	.11	^***^	.36	^***^	.02	^***^	-.33	^***^
Religious Attendance	2.17	1.41	2.30	1.63	.07	^***^	-.13	^***^	.54	^***^	.67	^***^
	.007	.006	.025	.012	.009		.026		.014		.028	
Religious Salience	5.18	4.24	5.71	4.91	.93	^***^	-.52	^***^	.27	^***^	.79	^***^
	.009	.010	.031	.015	.013		.033		.016		.033	
Age of First Alcohol Use	2.86	2.93	3.40	2.77	-.06	^***^	-.53	^***^	.09	^***^	.06	^***^
	.004	.003	.013	.007	.005		.014		.008		.014	
Lifetime Drug Use												
Tobacco	.56	.50	.50	.65	.06	^***^	.06	^***^	-.08	^***^	-.14	^***^
Cocaine	.14	.15	.06	.23	-.01	^***^	.07	^***^	-.09	^***^	-.17	^***^
Stimulants	.08	.12	.05	.12	-.04	^***^	.03	^***^	-.04	^***^	-.07	^***^
Sedatives	.08	.05	.08	.11	.02	^***^	-.00		-.03	^***^	-.03	^***^
Tranquilizer	.15	.15	.15	.20	.00		-.00		-.05	^***^	-.05	^***^
Inhalants	.07	.11	.02	.09	-.03	^***^	.05		-.02	^***^	-.07	^***^
Pain Relievers	.35	.34	.28	.39	.01	^*^	.06	^***^	-.04	^***^	-.10	^***^
Heroine	.01	.02	.01	.03	-.01	^***^	.00		.02	^***^	-.02	^***^
Marijuana	.42	.50	.20	.54	-.07	^***^	.22	^***^	-.11	^***^	-.33	^***^
PCP	.02	.02	.01	.01	.00	^***^	.01	^***^	-.02	^***^	-.03	^***^
MDMA/ecstasy	.04	.11	.01	.06	-.07	^***^	.03	^***^	-.02	^***^	-.05	^***^
Self-Reported Risky Behavior	1.54	1.86	1.34	1.58	-.32	^***^	.20	^***^	-.04	^***^	-.25	^***^
	.002	.002	.006	.004	.003		.007		.005		.008	

Source: 2008–2019 National Survey of Drug Use and Health, n = 674,521

^a^. Calculated with STATA LINCOM, Mean difference command

^b^. Standard deviations in parentheses.

^†^p < 0.10,

^*^p < 0.05,

^**^p < 0.01,

^***^p < 0.001 (two-tailed)

**Table 3:** Weighted multivariate ordinary least square regression pedicting the level of psychological distress in the past month.

	Model 1	Model 2	Model 3	Model 4	Model 5
Independent Variables					
LCPU	-0.1796^**^	-0.1777^**^	-0.0924	0.1824	-1.2117^***^
	(0.0666)	(0.0668)	(0.1005)	(0.2937)	(0.3347)
Marital Status ^a^					
Married		-0.7113^***^	-0.6893^***^	-0.7114^***^	-2.0956^***^
		(0.0633)	(0.0726)	(0.0633)	(0.2885)
Widowed		-0.5896^***^	-0.6274^***^	-0.5865^***^	-2.7136^***^
		(0.1200)	(0.1226)	(0.1200)	(0.5013)
Divorced		0.3635^***^	0.4280^***^	0.3636^***^	-1.1964^**^
		(0.0899)	(0.1046)	(0.0899)	(0.3997)
Household Size		0.4353^***^	0.4332^***^	0.4926^***^	0.0135
		(0.0723)	(0.0721)	(0.0761)	(0.1218)
Household Size Squared		-0.0251^*^	-0.0249^*^	-0.0355^**^	-0.0048
		(0.0105)	(0.0104)	(0.0111)	(0.0171)
Interaction Terms					
LCPU * Married			-0.1046		2.2372^**^
			(0.1381)		(0.8432)
LCPU * Widowed			0.5492		4.6197^***^
			(0.4695)		(1.2698)
LCPU * Divorced			-0.2755		2.0022^**^
			(0.2005)		(0.6841)
LCPU * Household Size				-0.3509	0.4678^*^
				(0.1959)	(0.2249)
LCPU * Household Size Squared				0.0640^*^	-0.0245
				(0.0285)	(0.0338)
Married * Household Size					0.5574^**^
					(0.1693)
Widowed * Household Size					1.2171^**^
					(0.4402)
Divorced * Household Size					0.8380^**^
					(0.2802)
Married * Household Size Squared					-0.0245
					(0.0235)
Widowed * Household Size Squared					-0.1021
					(0.0728)
Divorced * Household Size Squared					-0.0700
					(0.0423)
LCPU * Married * Household Size					-1.2178^*^
					(0.5273)
LCPU * Widowed * Household Size					-2.7436^**^
					(1.0340)
LCPU * Divorced * Household Size					-1.4114^**^
					(0.5093)
LCPU * Married * Household Size Squared					0.1267
					(0.0748)
LCPU * Widowed * Household Size Squared					0.3160
					(0.1642)
LCPU * Divorced * Household Size Squared					0.1724^*^
					(0.0782)
Constant	17.1953^***^	15.2715^***^	15.2781^***^	15.2181^***^	16.5710^***^
	(0.2038)	(0.2426)	(0.2419)	(0.2388)	(0.2749)
Observations	158633	158633	158633	158633	158633
*R* ^2^	0.146	0.152	0.152	0.152	0.154

Source: 2008–2019 National Survey of Drug Use and Health, n = 674,521

Standard errors in parentheses

^a^. Single Serves as the reference

^*^ *p* < 0.05,

^**^ *p* < 0.01,

^***^ *p* < 0.001

Note: Note: All models include controls for age, gender, race/ethnicity, income, full-time employment status, religious attendance, religious salience, lifetime drug use (cocaine, stimulants, sedatives, tranquilizers, heroine, pain killers, marijuana, PCP, ecstasy, inhalants, tobacco), age of first alcohol use, risky behaviors, and the survey year. Full models available in S1 Table

NSDUH created weights by adjusting the single-year weights by a scalar factor (i.e., the number of years of data used) so that the estimated number of individuals reported is representative of the national population. All analyses incorporate the sampling weights and complex study design provided by the NSDUH survey and conducted in STATA 17. The analysis presented all pooled data from 2008–2019. Except for testing the relationship between marital status, household size, and LCPU, this study replicates previous work that tested behaviors, including using the same control variables within logistic regressions [3, 10, 22, 25, 102] Those previous studies also pulled all available data in the NSDUH to run their analysis. Finally, as with previous research on psychedelics using the NSDUH, there was no control for multiple comparisons in the present study [18, 19, 28]. However, according to Armstrong [103], a Bonferroni correction is not needed for this study because it meets the following requirements: (1) it does not require a single test of the universal null hypothesis, (2) it does not need to avoid a type I error, and (3) it has extensive preplanned hypothesis that drive the analysis.

## Results

### Descriptive statistics

Table 2 presents the mean difference of weighted descriptive statistics by marital status. Married people had lower levels of distress compared to singles and divorced individuals (p < .001), but had similar levels of distress as those who were widowed. Widowed individuals had the lowest levels of LCPU, followed by married individuals, then singles, and finally divorcees (p < .001). Widowed individuals also had the smallest households, followed by divorcees, married individuals, and then singles (p < .001).

Married individuals were more likely to be Black, have a higher education, and have the highest level of income. On the other hand, widowed individuals had the lowest education levels, followed by singles, divorced individuals, and finally married individuals. Additionally, widowed individuals had the highest levels of religious attendance or salience (p < .001). Singles had the highest level of reported risky behavior, while widows had the lowest (p < .001). Singles were also the most likely to be fully employed.

Lastly, there were various differences in drug use, with one notable pattern being that divorcees had the highest levels of drug use across various substances, including tobacco, cocaine, tranquilizers, inhalants, pain relievers, heroin, marijuana, and they also started drinking at an earlier age (p < .001).

### Main effects of LCPU, marital status, and household size

Table 3 presents the results from the weighted ordinary least square regression. Model 1 shows that LCPU is associated with less psychological distress (b = -0.1796, p < .01), and this association remains significant in Model 2 when marital status and household size are added (b = -0.1777, p < .01). Model 2 reveals that being married (b = -0.7113, p < .001) or widowed (b = -0.5896, p < .001) is associated with lower levels of distress compared to being single, while being divorced is associated with higher levels of distress (b = 0.3635, p < .001). Furthermore, the quadratic equation shows that each one-unit increase in household size is associated with higher distress (b = 0.4353, p < .001), and this association decreases at an increasing rate (b = -0.0251, p < .05). Overall, these results confirm the prediction that LCPU is associated with less stress, larger households are associated with more stress, and being married is associated with lower levels of stress compared to being single.

### Two-way interactions

**Fig 1:**
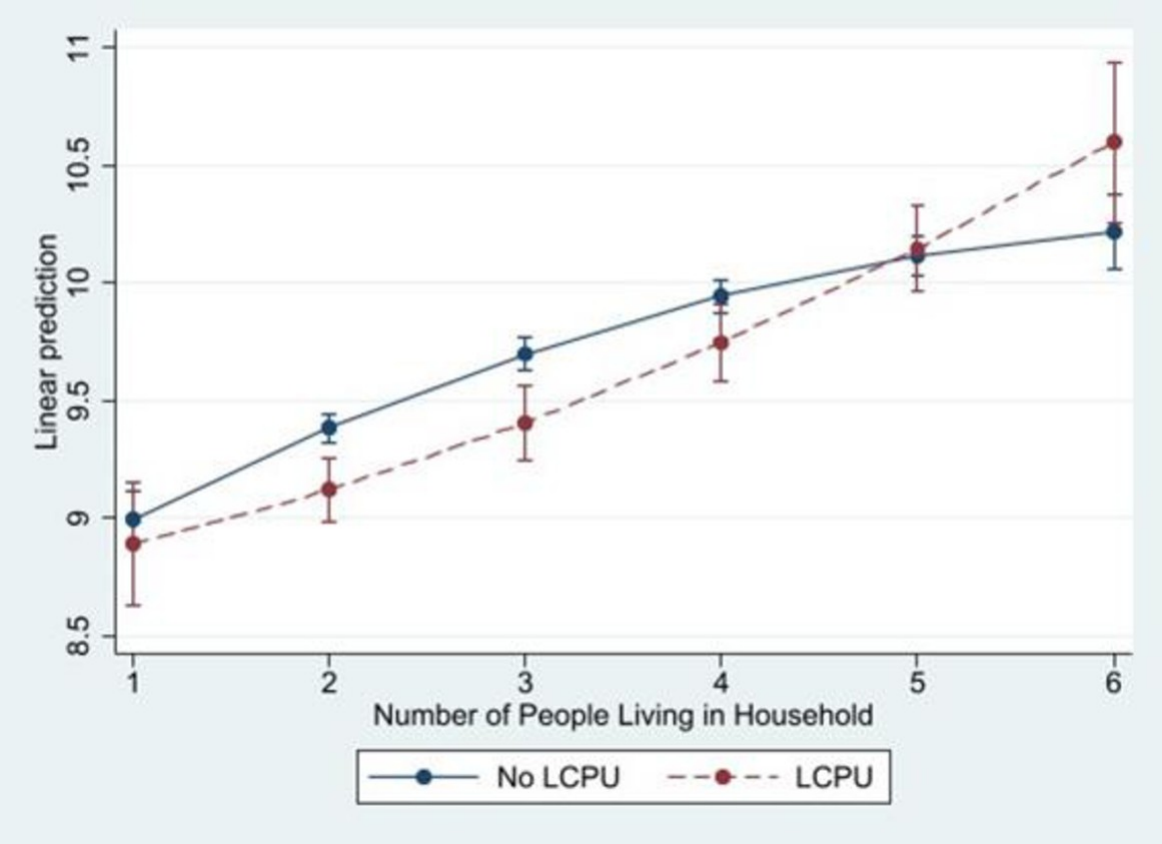
Predicted margins of LCPU* Household Size on the level of psychological distress with 95% Cis. Source: National Survey of Health and Social Behaviors, 2008–2019 Note: Based on Model 4, Table 3, multinomial OLS regression model predicting psychological distress in the past 30 days (k6).

Model 3 does not reveal an interaction between LCPU and marital status. However, the empirical prediction on the interaction between household size and LCPU was confirmed. Model 4 reveals that the coefficient for the curvilinear relationship between household size was significantly amplified by LCPU (b = 0.0640, p < .05). These results suggest that the positive association between household size and distress gets larger for each one-unit increase faster among those who have used psychedelics (Fig 1). Furthermore, marginal effects indicate that the interaction between LCPU and household size was not significant in one-person or four-person households. However, the interaction remained significant in two- and three-person households (p < .001), four-person households (p < .01), and six-person households (p < .05). In other words, as household size gets larger, the negative association of LCPU and health gets larger.

### Three-way interactions

Model 5 reveals multiple important relationships, including a three-way interaction between marital status, household size, and LCPU. First, the effects of LCPU are significantly diminished by being married (b = 2.2372, p < .01), widowed (b = 4.6197, p < .001), and divorced (b = 2.0022, p < .01) compared to being single. LCPU also attenuates the association between household size and distress (b = 0.4676, p < .05). Additionally, compared to singles, the effect of household size is significantly diminished among those who are married (b = 0.5574, p < .01), widowed (b = 1.2171, p < .001), and divorced (b = 0.8380, p < .01). Moreover, compared to single psychedelic users, the association between household size and distress is amplified among married (b = -1.2718, p < .05), widowed (b = -2.7436, p < .01), and divorced (b = -1.4114, p < .01) psychedelic users. Finally, the curvilinear relationship of household size is significantly increasing for divorced psychedelic users compared to single psychedelic users (b = 0.1724, p < .05).

**Fig 2:**
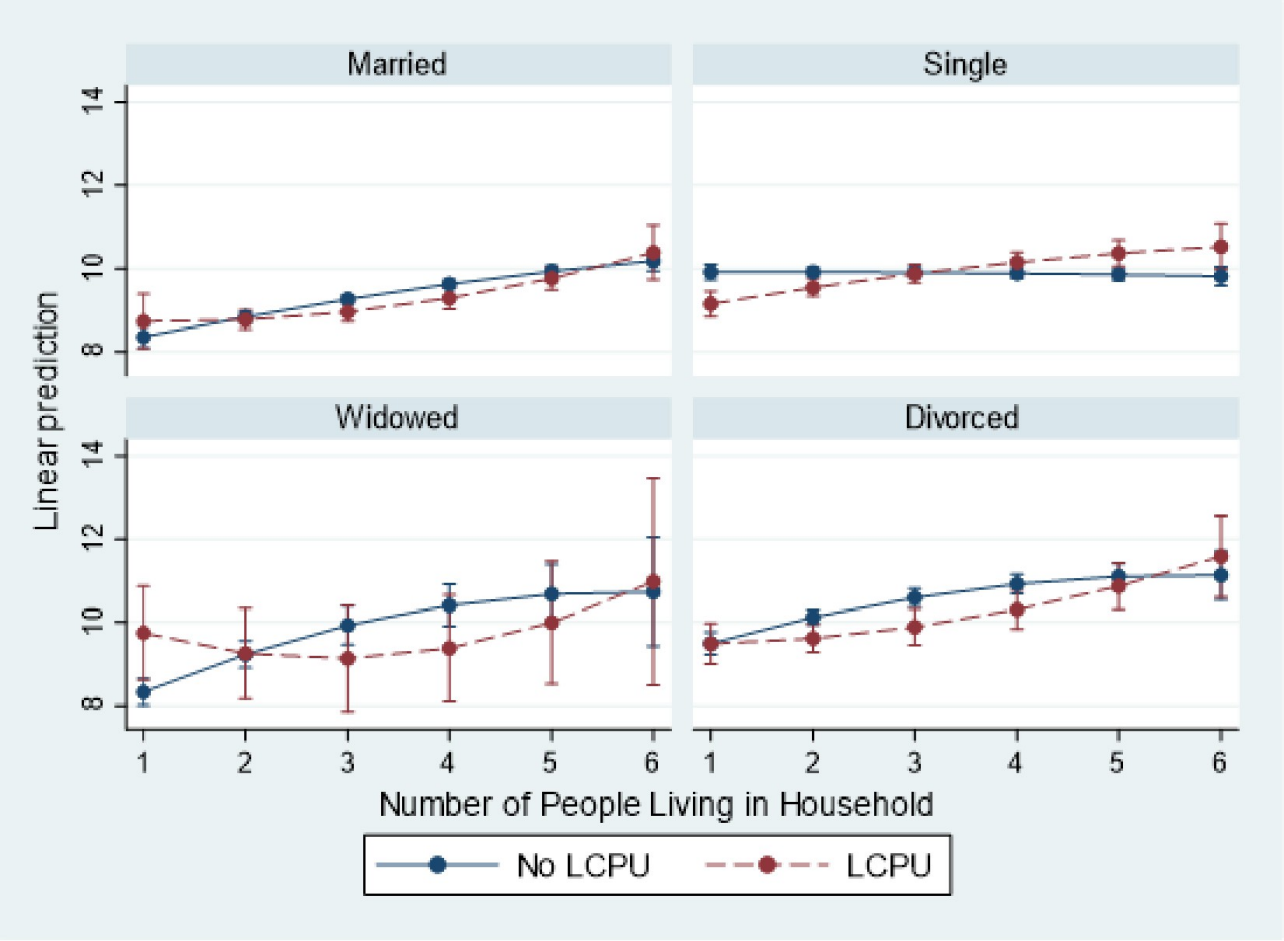
Predicted margins of LCPU * household Size by marital status on level of psychological distress with 95% Cis. Source: National Survey of Health and Social Behaviors, 2008–2019 Note: Based on Model 5, Table 3, multinomial OLS regression model predicting psychological distress in the past 30 days (k6).

Overall, the results confirm the predictions that LCPU exacerbates the negative consequences of household size for the heads of households who are married, widowed, and divorced. The results also suggest that larger households are associated with harm regardless of marital status, but the negative consequences decrease for single psychedelic users as the household size increases. Widowed psychedelic users may experience some benefits from living with more people, but these benefits decrease as the household size becomes too large. In contrast, among married or divorced psychedelic users, the distress caused by household size worsens as the family sizes increase. Finally, for widowed psychedelic users, there is a negative association between household size and distress, but this association decreases at a decreasing rate. These results can be explained by the increasing responsibilities that heads of households face as their families grow, which are then exacerbated by psychedelic use. On the other hand, single individuals may experience a diffusion of responsibility as their family sizes increase (Fig 2).

## Discussion

This study investigated the correlation between psychedelics, health, marital status, and household size. The impact of social integration and psychedelics on health and behaviors in the general population has not been extensively studied. As psychedelics become more accessible to the public, it is crucial to understand how cultural factors may influence the significance of these drugs. Building upon the modified theory of cultural set-and-setting, which suggests that certain subpopulations may experience diminished psychedelic effects, this study focused on two main questions: (1) Does marital status affect the effectiveness of psychedelics on health? (2) Does household size impact the efficacy of psychedelics on health? The study yielded several significant findings. The results confirmed the hypothesis that while low current psychedelic use (LCPU) is independently associated with better health, its interaction with larger households is linked to increased distress. Additionally, the findings highlighted the crucial role of social integration in the relationship between LCPU and health. The analysis provided partial evidence supporting the prediction that marital status predicts different health outcomes associated with psychedelic use, but this relationship is contingent upon the number of individuals residing in the household. In general, larger households tend to result in more distress and can weaken the positive association between LCPU and health, particularly for household heads. Furthermore, the study found that widowed individuals who use psychedelics may derive some benefits from living with more people, albeit not an excessive number.

Because the analysis utilizes marginal effects of the mean, it cannot determine the exact size at which households become harmful to health. However, the overall results suggest that any larger family is associated with worse distress among those who have used psychedelics. Why is there such a robust negative relationship between larger household size and health? Why does the intersection of household and LCPU lead to worse health? Two mechanisms may be at play. First, evidence suggests that larger families are generally associated with worse distress. People in larger households, despite having more social support, tend to experience chronic stress due to less privacy and louder, more disruptive environments [104–106]. Larger families also mean more responsibilities, which can be evenly distributed. However, when these responsibilities are not evenly distributed, they can have a significantly negative impact that is felt exponentially [107]. Rather than LCPU buffering the stress of larger households, the results suggest that LCPU may be exacerbating the negative effects of household size. Alternatively, those married people who have used psychedelics may be using them—and other substances—as a means to cope because they are experiencing more stress in larger households. In this way, the relationship is correlation; drug use is a product of stress, not necessarily an amplifier of it.

Importantly, the results did not find a direct interaction between LCPU and marital status alone. However, it was observed that the impact of LCPU on marital statuses varied depending on the size of the households. These variations could potentially be explained by the strength and nature of the relationships within these households, or the level of social integration experienced by individuals in different marital statuses. It is worth noting that certain studies have suggested that tightly-knit groups characterized by strict rules and regulations may lead to altruistic suicide, where individuals sacrifice themselves for the betterment of the community [49, 52, 53]. Results may be capturing heightened demands that are leading to compounding stress among heads of households who are holding families together. As the family size increases, so do the demands on the heads of households, and the likelihood that they are sacrificing their health for the good of the group. On the other hand, single people likely experience a diffusion of negative social regulation in larger households. While single individuals may be living with close families, those relationships may be more distant, resembling roommates as the family grows larger and there is less attention on any one individual. It is possible that singles in larger households can better disconnect from the stressors, while those who are married, divorced, and widowed are more closely tied to those negative pressures.

While larger households were generally found to be harmful to health, the study also revealed that widowed individuals who used psychedelics experienced a slight reduction in stress when living with a couple of other people. However, similar to other marital statuses, the positive effects of psychedelics diminished as the household size increased to three or more people. These findings align with previous research indicating that recently widowed individuals who intentionally surround themselves with close relationships are better able to cope with the stress of bereavement and social isolation [108]. Rather than being overwhelmed by numerous responsibilities in larger households, having a few close peers may serve as a buffer against stress, providing support and space for widowed individuals to potentially derive more benefits from psychedelics.

## Limitations and future directions

While this study uncovers a connection between marital status, household size, psychedelic use, and health, it does have some limitations. The primary limitation lies in the data itself. It is possible that individuals may still experience the benefits of psychedelic use regardless of their marital status or family size, but these benefits may diminish over time. To truly understand the impact of marital status, longitudinal data that includes the timing of drug use would be necessary.

Additionally, the analysis included a range of standard control sociodemographic variables, but it is likely that this list is not exhaustive. Previous findings in the field of psychedelic clinical trials suggest that other variables, such as personality traits, the presence and response to peak experiences, and dosage, could also influence outcomes. Furthermore, there may be unmeasured endogenous factors that contribute to the association between marital status, family, psychedelics, and distress. For example, while it is likely that married individuals bear more familial responsibilities as heads of the household, the analysis cannot confirm these specific relationships based on the available data.

Furthermore, the data is unable to parse out exact familial responsibility, the strength or happiness associated with relationships in the households, or the amount of relationship openness concerning stress, trauma, and drug use. As one of the first studies to statistically test the relationship between romantic relationships and family structures on psychedelics and health in a nationally representative sample, it is likely that other conditions—cultural and economic—may mitigate the positive effects of psychedelics. This study finds that social structure is critical for the relationship between psychedelics and health. Therefore, it is possible that, for instance, poverty, chronic stress, or even gendered reactions to assault and trauma, may also affect the salience of psychedelics. Future research should also test variation in marital status and household size by other important social statuses, including gender, race, and ethnicity, which evidence suggests affects familial roles, responsibility, and stress. Additionally, future research should also conceptualize perceptions of household sizes as a moderator. Specifically, some cultures (i.e., conservative Christian) may actually have a stronger preference for larger families [109, 110], which creates a different conceptual framework around the stress of familial responsibilities, thereby affecting the relationship between psychedelics and health.

The paper also identified significant patterns in marital status in relation to psychedelic use, which should be further investigated in future research. It is not surprising that singles exhibit a high level of psychedelic use, but it is noteworthy that divorced individuals have the highest usage. The disparity in psychedelic use between married and divorced individuals may be attributed to the characteristics of those who choose to remain in their marriages versus those who opt for dissolution. It is worth noting that there is a strong correlation between marital disillusionment and substance use [65, 111–113]. And even though widowed people arguably stand to gain a lot from psychedelic use as a means to cope with bereavement, results found that they had the lowest levels. This may be reflective of generational differences, as widowed people are older, and thus signals a potential barrier to providing them with care.

Most importantly, due to the cross-sectional study design, the results cannot be used to draw conclusive causal inferences, especially because we do not know the motivation behind the use of psychedelics. Those who use psychedelics in a clinical setting are likely doing so for health benefits, while those who use them in a naturalistic setting may have various motivations. This paper intentionally employs a modified cultural set and setting theoretical approach, which suggests that cultural differences and social inequality can greatly impact the effectiveness of certain drugs. By applying this enhanced theory, the aim of this work is to demonstrate disparities in outcomes associated with drug use by analyzing broader epidemiological patterns. This strategy is closely aligned with the research approach of this study. By establishing a strong foundation to highlight disparities at a macro level, this study paves the way for future investigations to examine these differences from a micro perspective. While I anticipate that a more detailed examination of the relationship between marital status and family on psychedelics would provide a more nuanced explanation, I do not expect it to substantially alter the results found in this paper, particularly regarding the importance of social integration for psychedelic experiences.

## Conclusion

Limitations notwithstanding, this study adds a critical new piece to the burgeoning research on psychedelics and health. One may ask why research should consider sociocultural conditions around the family from a population-level approach using a single lifetime use of classic psychedelics rather than those from clinical trials. First, these results represent how a population interacts with drugs in their everyday lives instead of in a controlled clinical environment. As psychedelics become more widely available, naturalistic use will rise faster than clinical treatment, especially in places where mental health treatment is severely underfunded. While family and romantic relationships are associated with different types and levels of stress, they also propel or deter mental health treatment, which will impact the overall impact of these medications on the community. Drawing on a modified theory of cultural set-and-setting, which argues that social conditions outside of the clinic will affect these drugs, those in relationships with more strain and fewer health resources, including time to cope, are not only likely to have access to clinical settings, but they also live in social situations that facilitate a severely compromised set-and-setting, leading to fewer positive outcomes even for naturalistic use.

Second and directly related, regardless of the motivation (clinical or recreational), psychedelics are associated with less distress and many other health benefits that are found in nationally represented samples. Population studies are worthwhile so that researchers can investigate whether this holds true among different populations and social statuses. The results suggest that society plays a more significant role in the effectiveness of psychedelics than some scholars may anticipate, especially among those who believe they can simply control away the effects of a community within the clinic. Since the dramatic shift toward clinical care, research has largely ignored the socio-cultural foundations of psychedelics. Important cultural processes have been abandoned, dismissed, and downplayed in subsequent psychedelic research. Psychedelic-assisted therapies carefully prime individuals prior to the psychedelic trip, including several months of therapy before treatment [4]. While studies on psychedelics can carefully create positive social relationships in clinical settings, they have yet to consider the impact of society once a person leaves the clinic. The results propose that several aspects of social integration could play a role. Marital status can affect psychedelic outcomes, but only when considering the familial setting people are living within. These results make it clear that more psychedelic use in itself may not actually lead to better outcomes, and a likely contributor to worse outcomes is higher stress in dense households. These results suggest that familial and social relationships outside of the clinical settings are important irrespective of treatment procedures. If people undergo psychedelic-assisted therapy that includes a positive relationship with counselors, those treatments may not produce the best outcomes, or worse, could exacerbate health problems if people do not have supportive family or social relationships at home.

## Supporting information

S1 Table Weighted multivariate ordinary least square regression predicting the level of psychological sistress in the past month. (DOCX)
