# Supplementary material for: Diminished psychedelic returns on distress: Marital status and household size
Source: PLoS One. 2024 Mar 7;19(3):e0293675. doi: 10.1371/journal.pone.0293675 (PMC10919602; doi:10.1371/journal.pone.0293675)
Supplement: S1 Table — (DOCX) [file pone.0293675.s001.docx]

| **S1 Table.** Weighted Multivariate Ordinary Least Square Regression Predicting the Level of Psychological Distress in the Past Month | | | | | |
| --- | --- | --- | --- | --- | --- |
|  | Model 1 | Model 2 | Model 3 | Model 4 | Model 5 |
| Independent Variables |  |  |  |  |  |
| LCPU | -0.1796^**^ | -0.1777^**^ | -0.0924 | 0.1824 | -1.2117^***^ |
|  | (0.0666) | (0.0668) | (0.1005) | (0.2937) | (0.3347) |
| Marital Status ^a^ |  |  |  |  |  |
| Married |  | -0.7113^***^ | -0.6893^***^ | -0.7114^***^ | -2.0956^***^ |
|  |  | (0.0633) | (0.0726) | (0.0633) | (0.2885) |
| Widowed |  | -0.5896^***^ | -0.6274^***^ | -0.5865^***^ | -2.7136^***^ |
|  |  | (0.1200) | (0.1226) | (0.1200) | (0.5013) |
| Divorced |  | 0.3635^***^ | 0.4280^***^ | 0.3636^***^ | -1.1964^**^ |
|  |  | (0.0899) | (0.1046) | (0.0899) | (0.3997) |
| Household Size |  | 0.4353^***^ | 0.4332^***^ | 0.4926^***^ | 0.0135 |
|  |  | (0.0723) | (0.0721) | (0.0761) | (0.1218) |
| Household Size Squared |  | -0.0251^*^ | -0.0249^*^ | -0.0355^**^ | -0.0048 |
|  |  | (0.0105) | (0.0104) | (0.0111) | (0.0171) |
| Interaction Terms |  |  |  |  |  |
| LCPU * Married |  |  | -0.1046 |  | 2.2372^**^ |
|  |  |  | (0.1381) |  | (0.8432) |
| LCPU * Widowed |  |  | 0.5492 |  | 4.6197^***^ |
|  |  |  | (0.4695) |  | (1.2698) |
| LCPU * Divorced |  |  | -0.2755 |  | 2.0022^**^ |
|  |  |  | (0.2005) |  | (0.6841) |
| LCPU * Household Size |  |  |  | -0.3509 | 0.4678^*^ |
|  |  |  |  | (0.1959) | (0.2249) |
| LCPU * Household Size Squared |  |  |  | 0.0640^*^ | -0.0245 |
|  |  |  |  | (0.0285) | (0.0338) |
| Married * Household Size |  |  |  |  | 0.5574^**^ |
|  |  |  |  |  | (0.1693) |
| Widowed * Household Size |  |  |  |  | 1.2171^**^ |
|  |  |  |  |  | (0.4402) |
| Divorced * Household Size |  |  |  |  | 0.8380^**^ |
|  |  |  |  |  | (0.2802) |
| Married * Household Size Squared |  |  |  |  | -0.0245 |
|  |  |  |  |  | (0.0235) |
| Widowed * Household Size Squared |  |  |  |  | -0.1021 |
|  |  |  |  |  | (0.0728) |
| Divorced * Household Size Squared |  |  |  |  | -0.0700 |
|  |  |  |  |  | (0.0423) |
| LCPU * Married * Household Size |  |  |  |  | -1.2178^*^ |
|  |  |  |  |  | (0.5273) |
| LCPU * Widowed * Household Size |  |  |  |  | -2.7436^**^ |
|  |  |  |  |  | (1.0340) |
| LCPU * Divorced * Household Size |  |  |  |  | -1.4114^**^ |
|  |  |  |  |  | (0.5093) |
| LCPU * Married * Household Size Squared |  |  |  |  | 0.1267 |
|  |  |  |  |  | (0.0748) |
| LCPU * Widowed * Household Size Squared |  |  |  |  | 0.3160 |
|  |  |  |  |  | (0.1642) |
| LCPU * Divorced * Household Size Squared |  |  |  |  | 0.1724^*^ |
|  |  |  |  |  | (0.0782) |
| Control Variables |  |  |  |  |  |
| Women | 0.9252^***^ | 0.8844^***^ | 0.8842^***^ | 0.8847^***^ | 0.8827^***^ |
|  | (0.0466) | (0.0474) | (0.0473) | (0.0473) | (0.0468) |
| Age | -0.5049^***^ | -0.4162^***^ | -0.4173^***^ | -0.4168^***^ | -0.4387^***^ |
|  | (0.0085) | (0.0103) | (0.0105) | (0.0103) | (0.0106) |
| Race ^b^ |  |  |  |  |  |
| Black | -0.3140^***^ | -0.4846^***^ | -0.4823^***^ | -0.4824^***^ | -0.4964^***^ |
|  | (0.0698) | (0.0700) | (0.0697) | (0.0700) | (0.0703) |
| Native American | 0.8260^**^ | 0.6689^*^ | 0.6741^*^ | 0.6601^*^ | 0.6645^*^ |
|  | (0.2581) | (0.2570) | (0.2580) | (0.2564) | (0.2615) |
| Hawaiian | 0.2678 | 0.0556 | 0.0603 | 0.0644 | 0.0535 |
|  | (0.4197) | (0.4159) | (0.4147) | (0.4163) | (0.4133) |
| Asian | 0.3402^***^ | 0.2595^**^ | 0.2625^**^ | 0.2625^**^ | 0.2486^*^ |
|  | (0.0962) | (0.0968) | (0.0966) | (0.0970) | (0.0953) |
| Multi-Racial | 0.3450^*^ | 0.2590 | 0.2604 | 0.2562 | 0.2617 |
|  | (0.1500) | (0.1519) | (0.1521) | (0.1517) | (0.1507) |
| Hispanic | 0.0437 | -0.1229 | -0.1242 | -0.1173 | -0.1192 |
|  | (0.0772) | (0.0780) | (0.0780) | (0.0783) | (0.0782) |
| Educational Attainment ^c^ |  |  |  |  |  |
| High School | -0.6389^***^ | -0.5833^***^ | -0.5853^***^ | -0.5837^***^ | -0.5878^***^ |
|  | (0.0745) | (0.0739) | (0.0740) | (0.0738) | (0.0741) |
| Some College | -0.6553^***^ | -0.6063^***^ | -0.6090^***^ | -0.6068^***^ | -0.6321^***^ |
|  | (0.0776) | (0.0771) | (0.0776) | (0.0774) | (0.0785) |
| College Degree or Higher | -1.2035^***^ | -1.0630^***^ | -1.0657^***^ | -1.0629^***^ | -1.1288^***^ |
|  | (0.0799) | (0.0787) | (0.0794) | (0.0790) | (0.0792) |
| Family Income | -0.3886^***^ | -0.3782^***^ | -0.3779^***^ | -0.3780^***^ | -0.3635^***^ |
|  | (0.0120) | (0.0122) | (0.0121) | (0.0122) | (0.0123) |
| Employed Full-Time | -0.3280^***^ | -0.3745^***^ | -0.3772^***^ | -0.3742^***^ | -0.4315^***^ |
|  | (0.0476) | (0.0477) | (0.0476) | (0.0478) | (0.0483) |
| Religious Attendance | -0.1442^***^ | -0.1329^***^ | -0.1328^***^ | -0.1332^***^ | -0.1327^***^ |
|  | (0.0148) | (0.0147) | (0.0147) | (0.0147) | (0.0145) |
| Religious Salience | -0.0494^***^ | -0.0509^***^ | -0.0508^***^ | -0.0511^***^ | -0.0500^***^ |
|  | (0.0112) | (0.0111) | (0.0111) | (0.0111) | (0.0110) |
| Drug Control Variables |  |  |  |  |  |
| Age of First Alcohol Use | -0.0471 | -0.0503 | -0.0502 | -0.0489 | -0.0443 |
|  | (0.0279) | (0.0274) | (0.0274) | (0.0275) | (0.0275) |
| Tobacco | 0.4645^***^ | 0.4539^***^ | 0.4528^***^ | 0.4530^***^ | 0.4552^***^ |
|  | (0.0550) | (0.0540) | (0.0538) | (0.0540) | (0.0540) |
| Cocaine | -0.0384 | -0.0947 | -0.0925 | -0.0922 | -0.0923 |
|  | (0.0669) | (0.0679) | (0.0689) | (0.0678) | (0.0694) |
| Stimulants | 0.6442^***^ | 0.6351^***^ | 0.6336^***^ | 0.6329^***^ | 0.6253^***^ |
|  | (0.0695) | (0.0689) | (0.0691) | (0.0689) | (0.0693) |
| Sedatives | 0.8253^***^ | 0.8420^***^ | 0.8456^***^ | 0.8446^***^ | 0.8578^***^ |
|  | (0.0715) | (0.0714) | (0.0715) | (0.0713) | (0.0709) |
| Tranquilizer | 1.0329^***^ | 1.0389^***^ | 1.0363^***^ | 1.0386^***^ | 1.0364^***^ |
|  | (0.0547) | (0.0543) | (0.0542) | (0.0543) | (0.0541) |
| Inhalant | 0.5619^***^ | 0.5492^***^ | 0.5526^***^ | 0.5508^***^ | 0.5471^***^ |
|  | (0.0725) | (0.0730) | (0.0727) | (0.0729) | (0.0732) |
| Pain Killer | 0.3847^***^ | 0.3684^***^ | 0.3668^***^ | 0.3686^***^ | 0.3532^***^ |
|  | (0.0598) | (0.0600) | (0.0602) | (0.0600) | (0.0601) |
| Heroine | 0.6972^***^ | 0.6416^***^ | 0.6349^***^ | 0.6373^***^ | 0.6325^***^ |
|  | (0.1485) | (0.1490) | (0.1487) | (0.1494) | (0.1493) |
| Marijuana | 0.4920^***^ | 0.4300^***^ | 0.4279^***^ | 0.4305^***^ | 0.4130^***^ |
|  | (0.0686) | (0.0695) | (0.0701) | (0.0696) | (0.0701) |
| PCP | -0.0382 | -0.0362 | -0.0242 | -0.0376 | -0.0161 |
|  | (0.1818) | (0.1811) | (0.1795) | (0.1811) | (0.1797) |
| MDMA | 0.2483^**^ | 0.2321^**^ | 0.2209^*^ | 0.2290^**^ | 0.2170^*^ |
|  | (0.0825) | (0.0833) | (0.0874) | (0.0832) | (0.0871) |
| Risky Behavior | 0.6797^***^ | 0.6702^***^ | 0.6692^***^ | 0.6703^***^ | 0.6698^***^ |
|  | (0.0313) | (0.0307) | (0.0308) | (0.0307) | (0.0307) |
| Survey Year ^d^ |  |  |  |  |  |
| 2009 | -0.0537 | -0.0634 | -0.0628 | -0.0642 | -0.0595 |
|  | (0.1203) | (0.1176) | (0.1176) | (0.1178) | (0.1160) |
| 2010 | 0.0513 | 0.0263 | 0.0267 | 0.0263 | 0.0364 |
|  | (0.1179) | (0.1147) | (0.1144) | (0.1148) | (0.1129) |
| 2011 | 0.0696 | 0.0406 | 0.0405 | 0.0409 | 0.0506 |
|  | (0.1163) | (0.1127) | (0.1126) | (0.1125) | (0.1116) |
| 2012 | 0.1595 | 0.1200 | 0.1195 | 0.1199 | 0.1276 |
|  | (0.1174) | (0.1144) | (0.1147) | (0.1147) | (0.1147) |
| 2013 | 0.2896^*^ | 0.2513 | 0.2497 | 0.2500 | 0.2680 |
|  | (0.1413) | (0.1377) | (0.1378) | (0.1379) | (0.1376) |
| 2014 | 0.1159 | 0.0795 | 0.0797 | 0.0790 | 0.0928 |
|  | (0.1046) | (0.1026) | (0.1024) | (0.1027) | (0.1019) |
| 2015 | -0.3027^*^ | -0.3425^**^ | -0.3416^**^ | -0.3405^**^ | -0.3174^**^ |
|  | (0.1172) | (0.1156) | (0.1154) | (0.1154) | (0.1143) |
| 2016 | -0.1914 | -0.2293^*^ | -0.2281^*^ | -0.2285^*^ | -0.2048 |
|  | (0.1109) | (0.1089) | (0.1085) | (0.1090) | (0.1072) |
| 2017 | -0.0391 | -0.0735 | -0.0724 | -0.0734 | -0.0527 |
|  | (0.1179) | (0.1151) | (0.1148) | (0.1151) | (0.1143) |
| 2018 | 0.2599^*^ | 0.2261 | 0.2261 | 0.2258 | 0.2431^*^ |
|  | (0.1216) | (0.1203) | (0.1200) | (0.1203) | (0.1186) |
| 2019 | 0.5779^***^ | 0.5451^***^ | 0.5437^***^ | 0.5444^***^ | 0.5635^***^ |
|  | (0.1262) | (0.1240) | (0.1241) | (0.1239) | (0.1223) |
| Constant | 17.1953^***^ | 15.2715^***^ | 15.2781^***^ | 15.2181^***^ | 16.5710^***^ |
|  | (0.2038) | (0.2426) | (0.2419) | (0.2388) | (0.2749) |
| Observations | 158633 | 158633 | 158633 | 158633 | 158633 |
| *R*^2^ | 0.146 | 0.152 | 0.152 | 0.152 | 0.154 |
| Source: 2008-2019 National Survey of Drug Use and Health, n= 674,521  Standard errors in parentheses  ^*^ *p* < 0.05, ^**^ *p* < 0.01, ^***^ *p* < 0.001  ^a^ Married serves reference category  ^b^ White, non-Hispanic services as the reference category  ^c^ Less than a high School degree serves as the reference category  ^d^ 2008 serves as the reference category | | | | | |
